# Supplementary material for: Imipenem Resistance Mediated by blaOXA-913 Gene in Pseudomonas aeruginosa
Source: Antibiotics (Basel). 2021 Sep 29;10(10):1188. doi: 10.3390/antibiotics10101188 (PMC8532623; doi:10.3390/antibiotics10101188)
Supplement: Supplementary file 1 [file antibiotics-10-01188-s001.zip › antibiotics-1354156-supplementary.pdf]

Supplementary tables

Table S1. Antimicrobial susceptibility profiles of *P. aeruginosa* K19PSE24.

Antimicrobial agent	Breakpoints (μg/mL)	Minimum Inhibitory Concentrations (μg/mL)
Amikacin	≥64	≤4
Ceftazidime	≥32	0.5
Ciprofloxacin	≥2	≤0.5
Colistin	≥4	≤2
Doripenem	≥8	2
Gentamicin	≥16	≤1
Imipenem	≥8	>8
Levofloxacin	≥4	≤0.5
Meropenem	≥8	2
Piperacillin/ Tazobactam	≥128/4	128/4
Ticarcillin/ Clavulanic Acid	≥128/2	16/2
Tobramycin	≥16	≤1

Table S2. Lists of primer used in the detection of carbapenem resistance genes in *P. aeruginosa*

Primers		Sequence	Size (bp)	References
bla$_{IMP}$	F	GGAATAGAGTGGCTTAAYTCTC	232	[11]
	R	GGTTTAAATAAAACAACCACC		
bla$_{VIM}$	F	GATGGTGTTTGGTCGCATA	390	[11]
	R	CGAATGCGCAGCACCAG		
bla$_{OXA\text{-}48\ like}$	F	TATATTGCATTAAGCAAGGG	800	[11]
	R	CACACAAATACGCGCTAACC		
bla$_{NDM}$	F	CACCTCATGTTTGAATTCGCC	984	[11]
	R	CTCTGTCACATCGAAATCGC		
bla$_{KPC}$	F	CATTCAAGGGCTTTCTTGCTGC	538	[12]
	R	ACGACGGCATAGTCATTTGC		

Abbreviations: F, forward; R, reverse

References

11. Borah, V.V.; Saikia, K.K.; Hazarika, N.K. First report on the detection of OXA-48-lactamase gene in *Escherichia coli* and *Pseudomonas aeruginosa* co-infection isolated from a patient in a tertiary care hospital in Assam. *Indian J Med Microbiol* **2016**, 34, 252–253.
12. El Garch, F.; Bogaerts, P.; Bebrone, C.; Galleni, M.; Glupczynski, Y. OXA-198, an acquired carbapenem-hydrolyzing class D-lactamase from *Pseudomonas aeruginosa*. *Antimicrob Agents Chemother* **2011**, 55, 4828–3483.
